# Supplementary material for: A cross-sectional study on COVID-19-related changes in self-medication with antibiotics
Source: PLoS One. 2022 Jun 14;17(6):e0269782. doi: 10.1371/journal.pone.0269782 (PMC9197057; doi:10.1371/journal.pone.0269782)
Supplement: S1 File — (DOCX) [file pone.0269782.s001.docx]

Gender 1female 0male

City 0big city 1smallcity 2countryside

Smoking 0no yes1

cause cheeckup 0 pain1 2 pus discharge, and abscess 3 dental trauma 4 tooth hypersensitivity 5 esthetic dental problems Drug no 0 yes1

Covid-19 0before 1 after

Drug name no 0 amoxicillin1 2 Co-amoxiclav Metronidazole 3 Azithromycin 4 5 Cefixime 6 Penicillin 7 Doxycycline 8 Clindamycin 9 wrong data
