# Supplementary material for: A cross-sectional study on COVID-19-related changes in self-medication with antibiotics
Source: PLoS One. 2022 Jun 14;17(6):e0269782. doi: 10.1371/journal.pone.0269782 (PMC9197057; doi:10.1371/journal.pone.0269782)

Overall a very good article relevant to the current situation during the pandemic. However, certain portions of the articles need to be rewritten for better clarity.

Prof. Datuk Dr Allan Mathews, PhD

PLOS ONE

COVID-19-related changes in the pattern of dental visits and self-medication with antibiotics

--Manuscript Draft--

Manuscript Number:	PONE-D-21-19078
Article Type:	Research Article
Full Title: A cross-sectional study on	COVID-19-related changes in the pattern of dental visits and self-medication with antibiotics
Short Title:	self-medication with antibiotics and COVID-19
Corresponding Author:	Dorsa Rahi<br>Guilan University of Medical Sciences<br>Rasht, IRAN, ISLAMIC REPUBLIC OF
Keywords:	Self-Medication, Anti-Bacterial Agents (suggest Antibiotics), Dentistry, COVID-19
Abstract:	Background and Aim: Dental treatments have been limited to emergency care due to the global rapid spread of coronavirus disease-2019 (COVID-19). Fear of contracting the disease in dental clinics has also altered the pattern of dental visits and self-medication. The present study compared the pattern of dental visits and self-medication with antibiotics (SMA) before and after the emergence of COVID-19 pandemic in a referral dental clinic in the north of Iran.<br>Materials and Methods: The data for the present cross-sectional study was collected from 756 patient records retrieved from the archives of the Faculty Clinic of Rasht School of Dentistry during two separate periods. In addition to demographic variables namely age, gender, and place of residence of patients, their smoking status, chief complaint, and SMA were also extracted from patient records. The Chi-square test and binary logistic regression models with 95% confidence interval served for statistical analysis.<br>Results: In total, 756 patient records (412 records from the pre-pandemic period and 344records from the post-pandemic period) were evaluated. SMA was significantly more prevalent after the pandemic compared to that before pandemic (OR= 3.39, 95% CI= 2.43-4.73, P=<0.001). The number of smoker patients significantly decreased after the pandemic by 6,6% compared to that in pre-pandemic period. Dental pain, pus discharge, and abscess as the chief complaints of patients were significantly more prevalent during the post-pandemic period; while, dental checkups, tooth hypersensitivity, and esthetic dental problems were significantly more frequent as the chief complaints of patients during the pre-pandemic period.<br>Conclusion: COVID-19 appears to increase SMA as well as the prevalence of acute dental problems in patients. With regard to the consequences of SMA such as antibiotic resistance, there ~~appears to be a~~ is the need to raise public awareness on this ~~topic~~ matter. Moreover, the public should be informed about the significance of early referral to dentists in order to prevent acute dental problems.<br>[Suggest reword - There is indication that during the Covid-19 pandemic, SMA iand prevalence of acute dental problems in patients.]
Order of Authors:	Dorsa Rahi
	Armin Gholamhossein Zadeh
	Mohammadreza Khami
Additional Information:	
Question	Response
**Financial Disclosure**<br><br>Enter a financial disclosure statement that describes the sources of funding for the work included in this submission. Review the submission guidelines for detailed requirements. View published research	This research had no funding source.

articles from *PLOS ONE* for specific examples.<br><br>This statement is required for submission and **will appear in the published article** if the submission is accepted. Please make sure it is accurate.<br><br>**Unfunded studies**<br>Enter: *The author(s) received no specific funding for this work.*<br><br>**Funded studies**<br>Enter a statement with the following details:<br>• Initials of the authors who received each award<br>• Grant numbers awarded to each author<br>• The full name of each funder<br>• URL of each funder website<br>• Did the sponsors or funders play any role in the study design, data collection and analysis, decision to publish, or preparation of the manuscript?<br>• **NO** - Include this sentence at the end of your statement: *The funders had no role in study design, data collection and analysis, decision to publish, or preparation of the manuscript.*<br>• **YES** - Specify the role(s) played.<br><br>* typeset	
**Competing Interests**<br><br>Use the instructions below to enter a competing interest statement for this submission. On behalf of all authors, disclose any competing interests that could be perceived to bias this work—acknowledging all financial support and any other relevant financial or non-financial competing interests.<br><br>This statement is required for submission and **will appear in the published article** if the submission is accepted. Please make sure it is accurate and that any funding sources listed in your Funding Information later in the submission form are also declared in your Financial Disclosure statement.	The authors have no conflict of interests to declare.

View published research articles from *PLOS ONE* for specific examples.<br><br>**NO authors have competing interests**<br><br>Enter: *The authors have declared that no competing interests exist.*<br><br>**Authors with competing interests**<br><br>Enter competing interest details beginning with this statement:<br><br>*I have read the journal's policy and the authors of this manuscript have the following competing interests: [insert competing interests here]*<br><br>* typeset	
**Ethics Statement**<br><br>Enter an ethics statement for this submission. This statement is required if the study involved:<br><br>• Human participants<br>• Human specimens or tissue<br>• Vertebrate animals or cephalopods<br>• Vertebrate embryos or tissues<br>• Field research<br><br>Write "N/A" if the submission does not require an ethics statement.<br><br>General guidance is provided below. Consult the submission guidelines for detailed instructions. **Make sure that all information entered here is included in the Methods section of the manuscript.**	The study protocol was reviewed and approved by the Ethics Committee of Tehran University of Medical Sciences (ID IR. TUMS.DENTISTRY.REC.1399.051), and written informed consent was obtained from patients.

Powered by Editorial Manager® and ProduXion Manager® from Aries Systems Corporation

**Format for specific study types**<br><br>**Human Subject Research (involving human participants and/or tissue)**<br>• Give the name of the institutional review board or ethics committee that approved the study<br>• Include the approval number and/or a statement indicating approval of this research<br>• Indicate the form of consent obtained (written/oral) or the reason that consent was not obtained (e.g. the data were analyzed anonymously)<br><br>**Animal Research (involving vertebrate animals, embryos or tissues)**<br>• Provide the name of the Institutional Animal Care and Use Committee (IACUC) or other relevant ethics board that reviewed the study protocol, and indicate whether they approved this research or granted a formal waiver of ethical approval<br>• Include an approval number if one was obtained<br>• If the study involved *non-human primates*, add *additional details* about animal welfare and steps taken to ameliorate suffering<br>• If anesthesia, euthanasia, or any kind of animal sacrifice is part of the study, include briefly which substances and/or methods were applied<br><br>**Field Research**<br><br>Include the following details if this study involves the collection of plant, animal, or other materials from a natural setting:<br>• Field permit number<br>• Name of the institution or relevant body that granted permission	
**Data Availability**<br><br>Authors are required to make all data underlying the findings described fully available, without restriction, and from the time of publication. PLOS allows rare exceptions to address legal and ethical concerns. See the PLOS Data Policy and FAQ for detailed information.	Yes - all data are fully available without restriction

A Data Availability Statement describing where the data can be found is required at submission. Your answers to this question constitute the Data Availability Statement and **will be published in the article**, if accepted.<br><br>**Important:** Stating 'data available on request from the author' is not sufficient. If your data are only available upon request, select 'No' for the first question and explain your exceptional situation in the text box.<br><br>Do the authors confirm that all data underlying the findings described in their manuscript are fully available without restriction?	
**Describe where the data may be found in full sentences. If you are copying our sample text, replace any instances of XXX with the appropriate details.**<br><br>• If the data are **held or will be held in a public repository**, include URLs, accession numbers or DOIs. If this information will only be available after acceptance, indicate this by ticking the box below. For example: *All XXX files are available from the XXX database (accession number(s) XXX, XXX.).*<br>• If the data are all contained **within the manuscript and/or Supporting Information files**, enter the following: *All relevant data are within the manuscript and its Supporting Information files.*<br>• If neither of these applies but you are able to provide **details of access elsewhere**, with or without limitations, please do so. For example:<br><br>*Data cannot be shared publicly because of [XXX]. Data are available from the XXX Institutional Data Access / Ethics Committee (contact via XXX) for researchers who meet the criteria for access to confidential data.*<br><br>*The data underlying the results presented in the study are available from (include the name of the third party*	Data is available upon request.

Powered by Editorial Manager® and ProduXion Manager® from Aries Systems Corporation

and contact information or URL).<br>• This text is appropriate if the data are owned by a third party and authors do not have permission to share the data.<br><br>* typeset	
Additional data availability information:	

Manuscript Click here to access/download;Manuscript;COVID manu.docx

A cross sectional study on ....

COVID-19-related changes in the pattern of dental visits and self-medication with antibiotics

Running title: self-medication with antibiotics and COVID-19

Dorsa rahi[1,2], Armin Gholamhossein Zadeh[1], Mohammadreza Khami[3,4*].

1. School of Dentistry, Guilan University of Medical Sciences, Rasht, Guilan, Iran.
2. Dental Sciences Research Center, School of Dentistry, Guilan University of Medical Sciences, Rasht, Iran.
3. School of Dentistry, Tehran University of Medical Sciences, Tehran, Iran.
4. Research Center for Caries Prevention, Dentistry Research Institute, Tehran University of Medical Sciences, Tehran, Iran.

*Corresponding author

Mohammadreza Khami: Assistant Professor of Community Oral Health.

Address: Amirabad Street, Tehran, Iran. Tel: +989123032747

Email: mkhami@razi.tums.ac.ir
https://orcid.org/ 0000-0002-4810-9537

Author contribution statements:

Conception or design of the work: D.Rahi , MR.Khami

Data collection: D.Rahi

Data analysis and interpretation: A.gholamhossein zade

Drafting the article: D.Rahi

Critical revision of the article: MR.Khami

Final approval of the version to be published: MR.khami

Data availability statement:

Data available on request from the authors. The data sets used and/or analysed during the current study are available from the corresponding author on reasonable request.

Acknowledgment

We acknowledge that all financial support and funding were provided by the Deputy of Research, International Campus, Tehran University of Medical Sciences.

Conflict of Interest

The authors have no conflict of interests to declare.

Ethics

The study protocol was reviewed and approved by the Ethics Committee of Tehran University of Medical Sciences (ID IR. TUMS.DENTISTRY.REC.1399.051), and written informed consent was obtained from patients.

Abstract

Background and Aim: Dental treatments have been limited to emergency care due to the global rapid spread of coronavirus disease-2019 (COVID-19). Fear of contracting the disease in dental clinics has also altered the pattern of dental visits and self-medication. The present study compared the pattern of dental visits and self-medication with antibiotics (SMA) before and after the emergence of COVID-19 pandemic in a referral dental clinic in the north of Iran.

Materials and Methods: The data for the present cross-sectional study was collected from 756 patient records retrieved from the archives of the Faculty Clinic of Rasht School of Dentistry during two separate periods. In addition to demographic variables namely age, gender, and place of residence of patients, their smoking status, chief complaint,
and SMA were also extracted from patient records. The Chi-square test and binary logistic regression models with 95% confidence interval served for statistical analysis.

Results: In total, 756 patient records (412 records from the pre-pandemic period
and 344records from the post-pandemic period) were evaluated. SMA was significantly more prevalent after the pandemic compared to that before pandemic (OR= 3.39, 95% CI= 2.43-4.73, P=<0.001). The number of ~~smoker patients~~ patients who smoke significantly decreased after the pandemic by 6,6% compared to that in pre-pandemic period. Dental pain, pus discharge, and abscess as the chief

complaints of patients were significantly more prevalent during the post-pandemic period; while, dental checkups, tooth hypersensitivity, and esthetic dental problems were significantly more frequent as the chief complaints of patients during the pre-pandemic period. **Conclusion:** COVID-19 appears to increase SMA as well as the prevalence of acute dental problems in patients. With regard to the consequences of SMA such as antibiotic resistance, there appears to be a need to raise public awareness on this topic. Moreover, the public should be informed about the significance of early referral to dentists in order to prevent acute dental problems.

See my suggestion of rewording in the first part of this document.

Maintain consistency and use Antibiotics

Keywords: Self-Medication, ~~Anti-Bacterial Agents~~, Dentistry, COVID-19

Introduction

The coronavirus disease-2019 (COVID-19) is an emerging infectious disease, rapidly evolving worldwide (1). World health organization (WHO) announcement on 11th of March 2020 ~~which~~ officially declares the spread of the COVID-19 virus as a global pandemic (2). It emerged unexpectedly, and has now turned into a challenging dilemma not only for the public, but also health professionals including dental practitioners, physicians, and medical and dental students worldwide (3). The American Dental Association released the list of emergency and non-emergency dental procedures for dental practitioners and the lay people, and emphasized on limiting the provision of dental services to emergency care only (4). China, as the first country reporting COVID-19 cases, mandated the use of personal protective equipment for all healthcare workers and stopped the provision of routine dental procedures (5). Such instructions should be strictly followed for prevention and control of COVID-19 until an effective vaccine or medication becomes available.

In Iran, all dental offices and clinics were shut down in the first 2 months following the announcement of COVID-19 pandemic by the WHO by the order of the Iranian Ministry of Health and Medical Education, and only some certain clinics, designated by the Ministry of Health remained open to provide emergency dental services. At the same time, safety protocols for dental care provision were compiled by the Ministry of Health for dental offices and clinics. Following relative subsidence of the COVID-19 cases in April 2020, the majority of the public and private dental clinics and offices resumed their routine activities, including the Faculty Clinic of Rasht School of Dentistry, located in Rasht, the capital city of Guilan Province in the northern part of Iran. Guilan province ranks second in terms of population density in Iran according to the 2016 census report (6). The Faculty Clinic of Rasht School of Dentistry accepts 100 patients daily, and is the largest dental clinic in Guilan Province, providing general and professional dental care services to the public. It is also the main referral dental clinic in Guilan Province.

In addition to the limitations set by the Ministry of Health, patients less commonly present to dental clinics and offices due to fear of contracting COVID-19. Resultantly, the patient flow and the complains of patients have greatly changed since the emergence of COVID-19. A retrospective study conducted in China on 2,537 patients reported a reduction in patient referrals to dental emergency care centers by 38% following the emergence of COVID-19. The number of female patients seeking dental care was higher than males, and the rate of oral and dental infections during this period reportedly increased from 51.0% to 71.9%. Fear of patients of contracting COVID-19 infection in dental offices further complications their dental problems, necessitating emergency care in some cases. It also elevates the risk of self-medication by patients (7).

Over 500 bacterial species colonize the oral cavity (8). The balanced combination of these microorganisms is referred to as the normal oral flora, which is required for oral health (9). According to a definition by the World Health Organization, self-medication refers to medication intake without a prescription, refilling old prescriptions, sharing medications with the

family members or one's social cycle, or using the available leftover medications (10). The reasons for self-medication may include limited access to healthcare facilities, shortage of healthcare services, illegal distribution of medications, wrong beliefs about physicians, and poor knowledge of individuals. Self-medication with antibiotics (SMA) has significant adverse effects such as drug toxicity, resistance of microorganisms, prolonged hospitalization, unsuccessful treatment, high cost of treatment, and increased rate of incurable diseases (11). In contrast to other medications and almost all other modalities, the efficacy of antibiotics is decreasing over time (12). A cross-sectional study of self-medication patterns of adults presenting to the dental clinic of Sharjah Dental School for their oral and dental problems revealed that 70.7% of patients had tried self-medication. The most common reason for self-medication was reported to be time shortage for visiting a dentist (37.6%), and not taking oral and dental problems seriously (36.8%) (13). SMA is a common problem in Iran. According to the available statistics, the rate of medication intake in Iran is three times the mean global rate (14, 15). A meta-analysis conducted from January 2000 to June 2019 on SMA by the university students in low- to moderate-income countries reported high prevalence of SMA; its prevalence was also correlated with level of education. Congo, Sudan, Ghana, Peru, Nispangeria, India, and Iran accounted for over 50% of SMA (16). Considering the increasing use of antibiotics, emergence of multi-drug resistant microorganisms is among the most important public health dilemmas (17). With regard to the alarming consequences of the possible increase in SMA among dental patients during the COVID-19 pandemic, the present study aimed to compare the pattern of dental visits and SMA before and after the emergence of COVID-19 pandemic in a referral dental clinic in the north of Iran.

Materials and Methods

Study design and participants:

This descriptive, cross-sectional study evaluated 756 records of patients referring to the Faculty Clinic of Rasht School of Dentistry during the morning shifts. The patients had been visited by a general dentist during a 6-month period, starting 3 months before the emergence of COVID-19

pandemic from mid-November 2019 to mid-February 2020, and after the emergence of COVID-19 pandemic from mid-April to mid-July 2020. The patient records were evaluated anonymously, and the extracted data were recorded in datasheets. The collected information is routinely recorded in patient records, and all patients had signed informed consent forms regarding the use of this information for research purposes. The study was approved by the Ethics Committee of Tehran University of Medical Sciences (IR.TUMS.DENTISTRY. REC.1399.136). Need this be in capitals??

The minimum sample size was calculated to be 350 for each time period considering 0.29 ratio (prevalence of SMA) in a previous study (18) before the emergence of COVID-19 and 0.42 ratio according to a pilot study, alpha=0.05, and d=0.13. The sample size was calculated according to the main objective of the study.

Data collection: Is there a Data Collection Form??

A general dentist evaluated patient records and retrieved the required information including the patients' gender, age, place of residence (capital city of the province, other urban areas, rural areas), smoking status, chief complaint, and SMA. Evaluation of patient records and extraction of information continued until the sample size for each time period was reached.

Statistical analysis:

Normal distribution of data was evaluated using the Shapiro-Wilk test, Kolmogorov-Smirnov test, or the kurtosis and skewness. Independent t-test, test and confidence interval for two proportions, and Chi-square test were used to find significant associations between the categorical independent (time before and after the COVID-19 pandemic) and dependent variables if the respective assumptions were met; otherwise, data were analyzed by the Mann Whitney test or the Fisher's exact test. The logistic regression model was applied to control for the effect of possible confounders. All statistical analyses were carried out by SPSS version 24 at 0.05 level of significance.

Restate to reflect true meaning

To rewrite to provide clarity

Results

In methodology stated minimum 350 for both groups.??

Of 756 patients included in this study, 412 (54.5%) were recruited from the pre-pandemic and 344 (45.5%) were recruited from the post-pandemic period. Of those recruited before the pandemic emergence, 195 (47.3%) were males. The mean age of patients recruited before the pandemic was 39.02 ± 13.84 years. Of those recruited after the pandemic emergence, 160 (46.5%) were males, and the mean age of these patients was 40.38 ± 15.91 years. The patients were homogeneous in terms of gender (p=0.822) and age (p=0.217) before and after the COVID-19 pandemic emergence (Table 1).

Table 1 shows that smoking, chief complaint, SMA, and the type of antibiotics taken ~~taken antibiotics~~ varied significantly before and after the pandemic emergence. The prevalence of smoking and the frequency of dental checkups, tooth hypersensitivity, and esthetic dental problems were ~~as~~ the chief complaints of patients decreased after the pandemic emergence compared to those before; whereas the frequency of dental pain, pus discharge, and abscess were ~~as~~ the chief complaints of patients increased after the pandemic emergence, compared to those before (Figure 1). SMA also significantly increased during ~~after~~ the pandemic, with amoxicillin and azithromycin being the most commonly used antibiotics.

rephrase for better clarity

separate sentences for Table 1 and Figure 1.

To evaluate the strength of the associations, simple and multiple logistic regression models were fitted to the data (Table 2).

Table 2 shows that the increase in SMA was significant after the pandemic emergence in both unadjusted and adjusted models, and the odds of taking antibiotics after the pandemic emergence increased by 3.39 (95%CI=2.43-4.73) times. The change in smoking status, which was significant in the unadjusted model, remained non-significant in the adjusted model. The odds of referral due to dental pain, pus discharge and abscess, tooth fracture, tooth hypersensitivity and checkup increased by 11.19 (95%CI=4.89-25.59), 10.99 (95%CI=4.75-25.45), 7.75 (95%CI=3.03-19.82) and 3.23 (95%CI=1.19-8.77) times, respectively.

Discussion

According to the results, the rate of SMA increased by 30% after the pandemic emergence, compared to that before. Considering the complications and adverse consequences of SMA, and the fact that SMA is a major public health concern, this is an alarming finding that calls for attention of dental clinicians and healthcare personnel, and highlights the need for public awareness on this topic. The increased rate of SMA appears to be due to the postponement of dental visits by patients as the result of fear of contracting COVID-19, leading to self-medication. On the other hand, late seeking of dental care would further complicate the problem and increase the rate of emergency cases (4, 19), which was also highlighted in the present study since the results showed a shift in the chief complaints of patients from non-emergency dental problems before the pandemic to emergency problems after the pandemic emergence.

Of self-medicated antibiotics, azithromycin and amoxicillin had been more commonly used compared to others. Amoxicillin is commonly prescribed by dental practitioners (20). Self-medication with azithromycin is probably related to the primary assumptions of patients regarding he effectiveness of this antibiotic against the coronavirus (21).

No significant correlation was noted between SMA and place of residence (urban versus rural areas) or age of patients in the present study. Gender had no significant correlation with SMA before or after the pandemic emergence. Also, the referral rate of male and female patients was almost the same in the present study while Radeva et al. reported higher referral rate of females both before and after the pandemic emergence (22). Another study on utilization of dental services also reported higher utilization of dental services by females (23).

The chief complaints of patients mainly included dental checkup, esthetic dental problems, tooth discoloration, and tooth hypersensitivity before the pandemic emergence; while, the chief complaints mainly included dental pain, abscess, and tooth fracture after the pandemic emergence. This finding indicates that patients sought dental care for more serious and more acute conditions after the pandemic emergence due to the fear of contracting COVID-19 and the

set restrictions (24). In addition to home quarantine, depression, fear, and anxiety related to COVID-19 pandemic have resulted in an increase in the prevalence of hysterical dental pain. Also, home quarantine and greater consumption of sugary substances have aggravated the pattern of dental caries (25). Another reason might be the lack of patient information regarding provision of non-emergency dental care services during the pandemic period, resulting in higher percentage of emergency cases.

Ref??

In a similar study by Radeva, of patients seeking dental care during the pandemic period, 41% had tooth or restoration fracture, 20% had irreversible pulpitis, 18% had periodontal disease, 15% required prosthodontic treatment, and 6% had symptomatic or asymptomatic. Of patients seeking dental care before the pandemic emergence, 39% had apical periodontitis, 33% had dental caries and required restoration, 13% had irreversible pulpitis, 9% had periodontal disease, and 6% required prosthodontic treatment (22). These findings highlight the greater significance of patient education, prevention, and oral health promotion during the pandemics, and dental practitioners should more actively participate in preventive programs and oral health instruction during the COVID-19 pandemic and possible future pandemics.

Check spacing of paragraphs.

In the present study, a significant correlation was noted between cigarette smoking and SMA. Smokers visited the dental clinics less frequently than non-smokers after the COVID-19 pandemic emergence; this finding may be due to higher susceptibility of smokers to respiratory diseases and the COVID-19 (26, 27).

The present study was carried out in a large professional dental clinic, which is a referral center for all types of oral and dental conditions in a large city in Iran. Also, all the information was extracted and recorded by one dentist, which was another strength of this study. However, the cross-sectional design was the main limitation of the present study.

Conclusion

The increased frequency of SMA for dental problems during the COVID-19 pandemic, and its role in emergence of bacterial resistance and subsequent adverse effects on the immune

system highlight the critical role of dental practitioners in raising public awareness on this topic. Pharmacists can also play a role in this respect by not providing prescription drugs to patients without a prescription and correct guidance of patients to seek dental treatment. Physicians can also help in this regard by enhancing the public knowledge in this respect. Last but not least, dental practitioners should have updated knowledge about pharmacology to take a step forward in decreasing the rate of microbial resistance.

References

1. Meshkat S, Salimi A, Joshaghanian A, Sedighi S, Sedighi S, Aghamollaii V. Chronic neurological diseases and COVID-19: Associations and considerations. Translational neuroscience. 2020;11(1):294-301. Web of Science
2. Zhang D, Hu M, Ji Q. Financial markets under the global pandemic of COVID-19. Finance Research Letters. 2020;36:101528. Web of Science
3. Spagnuolo G, De Vito D, Rengo S, Tatullo M. COVID-19 outbreak: an overview on dentistry. International Journal of Environmental Research and Public Health. 2020;17(6):2094. Web of Science
4. Ather A, Patel B, Ruparel NB, Diogenes A, Hargreaves KM. Coronavirus disease 19 (COVID-19): implications for clinical dental care. Journal of endodontics. 2020;46(5):584-95. Web of Science
5. Meng L, Hua F, Bian Z. Coronavirus disease 2019 (COVID-19): emerging and future challenges for dental and oral medicine. Journal of dental research. 2020;99(5):481-7. Web of Science
6. Falahati S, Azizi J. Role of Rural Markets in Rural People's Economic Prosperity in Guilan Province of Iran. Village and Development. 2019;22(2):125-39.
7. Guo H, Zhou Y, Liu X, Tan J. The impact of the COVID-19 epidemic on the utilization of emergency dental services. Journal of dental sciences. 2020. Web of Science
8. Reynolds-Campbell G, Nicholson A, Thoms-Rodriguez C-A. Oral bacterial infections: Diagnosis and management. Dental Clinics. 2017;61(2):305-18.
9. Goodson JM, Hartman M-L, Shi P, Hasturk H, Yaskell T, Vargas J, et al. The salivary microbiome is altered in the presence of a high salivary glucose concentration. PloS one. 2017;12(3):e0170437. Web of Science
10. Organization WH. Guidelines for the regulatory assessment of medicinal products for use in self-medication. World Health Organization; 2000.
11. Aslam A, Gajdács M, Zin CS, Binti Abd Rahman NS, Ahmed SI, Jamshed SQ. Public awareness and practices towards self-medication with antibiotics among the Malaysian population. A development of questionnaire and pilot-testing. Antibiotics. 2020;9(2):97. Web of Science

12. Ateshim Y, Bereket B, Major F, Emun Y, Woldai B, Pasha I, et al. Prevalence of self-medication with antibiotics and associated factors in the community of Asmara, Eritrea: a descriptive cross sectional survey. BMC public health. 2019;19(1):1-7.
13. AlQahtani HA, Ghiasi FS, Zahiri AN, Rahmani NI, Abdullah N, Al Kawas S. Self-medication for oral health problems among adults attending the University Dental Hospital, Sharjah. Journal of Taibah University Medical Sciences. 2019;14(4):370-5. Web of Science
14. Jafari F, Khatony A, Rahmani E. Prevalence of self-medication among the elderly in Kermanshah-Iran. Global journal of health science. 2015;7(2):360.
15. Ruiz ME. Risks of self-medication practices. Current drug safety. 2010;5(4):315-23.
16. Xu R, Mu T, Wang G, Shi J, Wang X, Ni X. Self-medication with antibiotics among university students in LMIC: a systematic review and meta-analysis. The Journal of Infection in Developing Countries. 2019;13(08):678-89.
17. Guerrini L, Monaco A, Pietropaoli D, Ortu E, Giannoni M, Marci MC. Antibiotics in dentistry: a narrative review of literature and guidelines considering antibiotic resistance. The Open Dentistry Journal. 2019;13(1).
18. Karimi M, Maghsoodi E, Zayer B, Rashkeie S, Zareie F. The Frequency of Self-medication and Its Effective Factors in Students and Their Peer Group in the City of Boukan in 2016: A Descriptive Study. Journal of Rafsanjan University of Medical Sciences. 2019;18(8):753-68.
19. Staub M, Beaulieu R, Graves J, Nelson G. Changes in Antimicrobial Utilization During the COVID-19 Pandemic after Implementation of a Multispecialty Clinical Guidance Team. Infection Control and Hospital Epidemiology. 2020:1-28.
20. El-Kholey KE, Wali O, Elkomy A, Almozayen A. Pattern of antibiotic prescription for oral implant treatment among dentists in Saudi Arabia. Implant dentistry. 2018;27(3):317-23. Web of Science
21. da Silva CF, Deutschendorf C, Nagel FM, Dalmora CH, Dos Santos RP, Lisboa TC. Impact of the pandemic on antimicrobial consumption patterns. Infection Control & Hospital Epidemiology. 2020:1-3.
22. Radeva EN. Characteristics of Dental Treatment in two months' Quarantine due to Coronavirus Disease (COVID-19).
23. Šiljak S, Janković J, Marinković J, Erić M, Janevic T, Janković S. Dental service utilisation among adults in a European developing country: findings from a national health survey. International dental journal. 2019;69(3):200-6. Web of Science
24. Brkić H. Dental medicine and COVID-19 pandemic. Acta Stomatologica Croatica. 2020;54(2):118. Web of Science
25. Campagnaro R, de Oliveira Collet G, de Andrade MP, Salles JPdSL, Fracasso MdLC, Scheffel DLS, et al. COVID-19 pandemic and pediatric dentistry: Fear, eating habits and parent's oral health perceptions. Children and youth services review. 2020;118:105469. Web of Science
26. Berlin I, Thomas D, Le Faou A-L, Cornuz J. COVID-19 and smoking. Nicotine and Tobacco Research. 2020;22(9):1650-2. Web of Science
27. El-Zoghby SM, Soltan EM, Salama HM. Impact of the COVID-19 pandemic on mental health and social support among adult Egyptians. Journal of community health. 2020;45:689-95. Web of Science

Table 1. Comparisons of the studied variables before and after the COVID-19 pandemic emergence among a group of dental patients (n=756) in Iran

	Time		**P-value ()**
	Before the pandemic n(%)	After the pandemic n(%)	
**Gender**			
Male	195 (47.3)	160 (46.5)	0.822 (0.05)
Female	217 (52.7)	184 (53.5)	
**Place of residence**			
Capital city	229 (55.6)	214 (62.2)	0.142 (3.90)
Urban areas	113 (27.4)	75 (21.8)	
Rural areas	70 (17)	55 (16)	
**Smoking status**			
Non-smoker	319 (77.4)	289 (84)	0.023 (5.16)
Smoker	93 (22.6)	55 (16)	
**Chief complaint**			
Checkup	70 (17)	7 (2)	<0.001 (115.43)
Pain	123 (29.9)	166 (48.3)	
Pus and abscess	93 (22.6)	124 (36)	
Fracture	36 (8.7)	30 (8.7)	
Hypersensitivity	39 (9.5)	16 (4.7)	
Esthetic problems	51 (12.4)	1 (0.3)	

**Self-medication with antibiotics**			
No	303 (73.5)	151 (43.9)	<0.001 (68.69)
Yes	109 (26.5)	193 (56.1)	
**Medication**			
None	303 (73.5)	151 (43.9)	<0.001 (80.57)
Amoxicillin	44 (10.7)	78 (22.7)	
Co-amoxiclav	11 (2.7)	13 (3.8)	
Metronidazole	8 (1.9)	8 (2.3)	
Azithromycin	3 (0.7)	16 (4.7)	
cefixime	3 (0.7)	3 (0.9) td style="width:101.9pt; padding-right:5.4pt; padding-left:5.4pt; vertical-align:middle; background-color:#ffffff">	
Penicillin	7 (1.7)	6 (1.7)	
Doxycycline	4 (1)	3 (0.9)	
Clindamycin	1 (0.2)	2 (0.6)	
Incomplete information	19 (4.6)	51 (14.8)	
Amoxicillin & Metronidazole	9 (2.2)	12 (3.5)	
Azithromycin & Penicillin	0 (0)	1 (0.3)	

Table 2. Changes in SMA*, smoking status, and chief complaints of patients after the COVID-19 pandemic emergence, compared with before using simple (unadjusted) and multiple (adjusted) logistic regression models among a group of dental patients (n=756) in Iran

		**OR (95% CI)**			
		Unadjusted	P value	Adjusted	Pvalue
**SMA***	No	1			
	Yes	3.55 (2.62 - 4.82)	<0.001	3.39 (2.43 – 4.73)	<0.001
**Smoking**	Non-smoker	1.53 (1.06 - 2.22)	0.024	1.48 (0.97 – 2.24)	0.066
	Smoker	1			
**Chief complaint**	Checkup	1			
	Dental pain	13.50 (6 - 30.37)	<0.001	11.19 (4.89 – 25.59)	<0.001
	Pus and abscess	13.33 (5.86 – 30.34)	<0.001	10.99 (4.75 – 25.45)	<0.001
	Fracture	8.33 (3.33 – 20.82)	<0.001	7.75 (3.03 – 19.82)	<0.001
	Hypersensitivity	4.10 (1.55 – 10.83)	0.004	3.23 (1.19 – 8.77)	0.022
	Esthetic problems	0.20 (0.02 – 1.64)	0.133	0.15 (0.02 – 1.25)	0.079

*** Self-medication with antibiotics**

Figure 1. **Changes in the chief complaints of patients after the COVID-19 pandemic emergence compared with before among a group of dental patients (n=756) in Iran**

Figure

Click here to access/download;Figure;IMG_6684.jpg

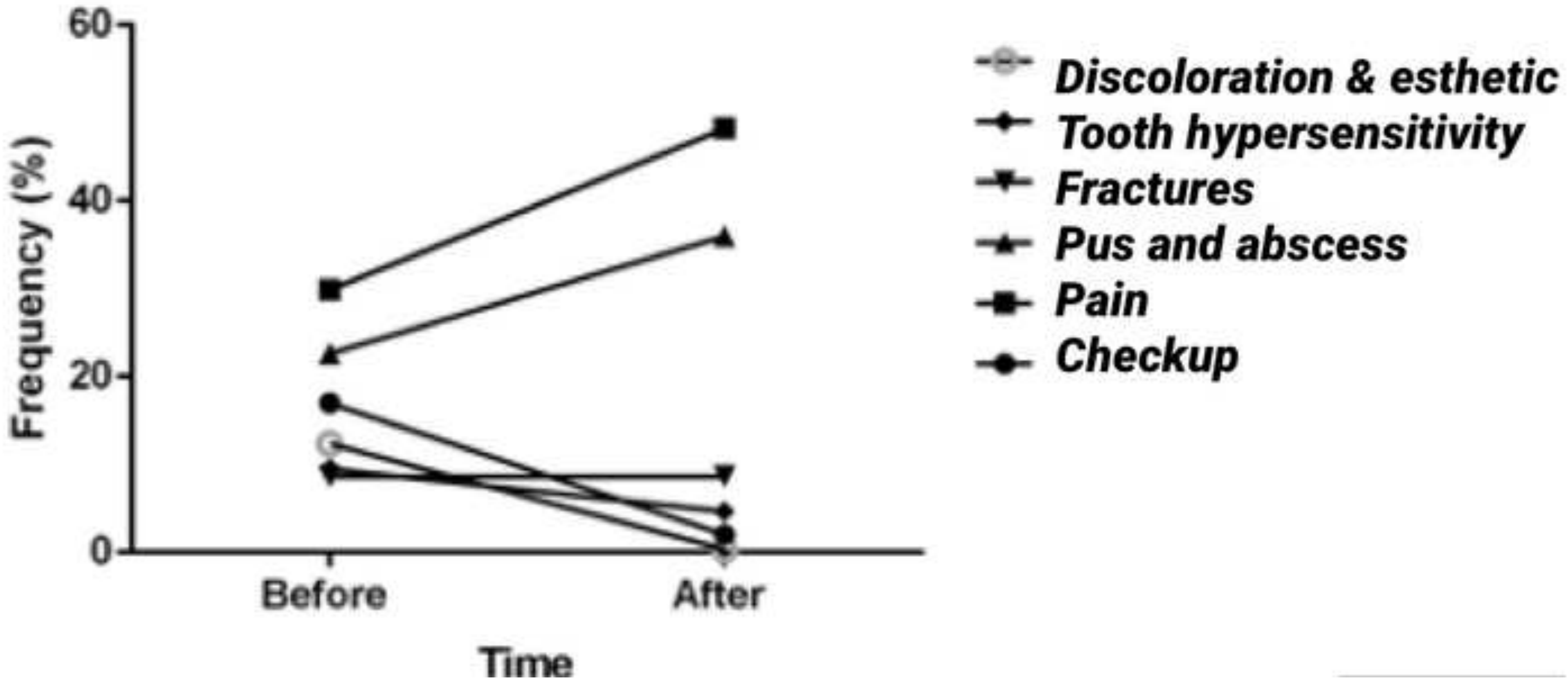

Supplement: Attachment — Submitted filename: PONE-D-21-19078_reviewer.pdf [file pone.0269782.s004.pdf]
