# Supplementary material for: A cross-sectional study on COVID-19-related changes in self-medication with antibiotics
Source: PLoS One. 2022 Jun 14;17(6):e0269782. doi: 10.1371/journal.pone.0269782 (PMC9197057; doi:10.1371/journal.pone.0269782)
Supplement: Attachment — Submitted filename: Response-Reviewer 2.docx [file pone.0269782.s003.docx]

Reviewer #2:

Abstract

With great regards, we kindly appreciate your time and accurate comments. Your comments help us to improve our manuscript. We revised the manuscript according to the comments, as explained below.

1- In results, line no. 4 there would be 6.6 rather than 6,6.

Response: 6,6 was revised to 6.6 (Page 1, line 20)

2- Conclusion should be based on the results of the study. Was the association between antibiotic resistance and SMA assessed in your study?

Response: The association between antibiotics resistance and SMA was not assessed in this study, and the conclusion was revised (Page 2, line 4,5,6).

3- P=<0.001? this is not the correct way to write significance.

Response: The P value was corrected (Page 1, line 19).

Introduction
4- Introduction seems to lengthy. It is better to make it to-the-point and crisp.

Response: We revised and shortened the introduction (Pages 2,3,4).

5- World health organization should be World Health Organization.
Response: The correction was done (Page 2 line10).

6- In the first paragraph, “and” is written repeatedly line 5 & 7.
Response: The corresponded sentences were revised (Page 2, lines 5 and 7).

7- In paragraph 3, second last line, it should be complicates rather than complications.
Response: Complications was revised to complicates (Page 3, line 5).

Methodology
8- Based on the fact that it is a pre and post COVID-19 study, please explain the study design.
Response: Thank you for your valuable comment. The study design was revised to cross sectional study (Page 4, line 10).

9- I will suggest that the study setting should be part of methods section rather than the introduction section.

Response: Thank you for your suggestion. We added the study setting to methods, and removed it from introduction

10- How the authors approached the participants to take their written consent specifically for post COVID-19 patients?
Response: There is a routine statement in the hospital records, requiring the patients to show if they give consent to use their data anonymously for research purposes. The patients have the option to deny. In our study, all the patients’ had shown agreement to use their data for research purposes. This was considered as written informed consent. (Page 4, line 15-19).

11- Please explain if the sample size calculation formula is correct for cross sectional studies.
 Since our main outcome was the proportion of the patients with SMA, we used the following formula, which uses proportions to calculate sample size in cross sectional studies. We used an estimated proportion of SMA from previous studies.

$$n=\frac{\left(z_{1-\frac{\alpha}{2}}\right)^{2}.p(1-p)}{d^{2}}=\frac{\left(1.96\right)^{2}0.206(1-0.206)}{(0.0412)^{2}}=370.17\cong371$$

12- What was the sampling strategy?
 Any patient referring to the Faculty Clinic of Rasht School of Dentistry during the morning shifts and was older than 18 years old was included in the study.” (Page 4, line 10,11)

13- In data collection section, what about the sampling criteria?
Response: Inclusion criteria is added to the method. “Any patient referring to the Faculty Clinic of Rasht School of Dentistry during the morning shifts and was older than 18 years old was included in the study.” (Page 4, line10,11, and page 5, lines 8 and 9)

14- Please follow the standard format of writing SPSS.
Response: Revised to “SPSS version 24 (IBM Corp, Armonk, NY, USA)”

Results
15- In the Table 1, what P() column refers to?
Response: The P refers to p value and X^2^  refers to the Chi-square value.

16- Table 2 is not in the standard format to present result of logistic regression analysis. It is equally not clear from the Table that what is the dependent variable. Further, Table 2 does not justify the use of logistic regression analysis to show the variation in SMA before and after COVID-19 era.

Response: Thank you for your valuable comment. The table 2 was revised substantially. Now the dependent variable is SMA, and the data presentation format has been revised (Pages 9,10).

17-Overall, the results section needs a major re-write.
The results part has now been revised (Pages 9,10).

Discussion and conclusion
18- I would suggest healthcare professionals rather than personnel.
Response: The healthcare professional was changed to personnel (Page10, line9).

19- Paragraph 2, second last line “the” instead of “he.”
Response: Revised. We kindly appreciate your accuracy.

20- Can you please give the list of self-medicated antibiotics other than amoxicillin and azithromycin?
Response: Revised to “Of self-medicated antibiotics (including Amoxicillin, Co-amoxiclav, Metronidazole, Azithromycin, Cefixime, Penicillin, Doxycycline and Clindamycin), Azithromycin and Amoxicillin had been more commonly used compared to others” (Page11, line3,4).

21- Overall, the Discussion part should be improved and elaborated

Response: The Discussion part has now been revised and elaborated substantially, with the main focus on SMA (Pages:10,11,12)

22- Conclusion section needs improvement and should be coherent with the results. The recommendations made in the conclusion section should be aligned with the results and main objectives of the study.

Response: We revised Conclusion part to address this comment (Page13, line1-5).
