# Supplementary material for: A cross-sectional study on COVID-19-related changes in self-medication with antibiotics
Source: PLoS One. 2022 Jun 14;17(6):e0269782. doi: 10.1371/journal.pone.0269782 (PMC9197057; doi:10.1371/journal.pone.0269782)
Supplement: Attachment — Submitted filename: Point by point response to the reviewer.docx [file pone.0269782.s005.docx]

We would like to cordially thank the reviewer for the constructive comments. Here is our point by point responses to the comments

1. the add "A cross-sectional study on............." to the title to give a reader the full picture.

Response: The title was revised according to the comment.
2. rewording of some parts of the write-up to provide clarity (pl refer to the reviewed article)

Response: All requested rewordings were done.

3. Details of Data Collection Form to be included in the write-up.

Response: A sentence on data collection form was added to the paragraph on data collection (page 5). The data collection form included the variables explained in the same paragraph.

4. There is a mis-match of the number of respondents - 350 required and only 344 studied?

Response: We collected the data from all available complete records in the specified time periods. The number of these records in the post-pandemic period was 344. The biostatistician author confirmed that this will not affect the analyses and results.

5. Table 1 and Figure 1 descriptions to be separated.

Response: The results part was revised to address this comment (page 8, first paragraph).

Comments inserted in the text:

Suggest reword - There is indication that during the Covid-19 pandemic, SMA and prevalence of acute dental problems.

Response: The conclusion in the abstract and in the main text was revised according to the comment.

“patients who smoke” in the results of abstract.

Response: The results of abstract was revised.

Maintain consistency and use Antibiotics (in the keywords).

Response: Revised

“complicates” (in the introduction)

Response: Revised.

Need this be in capitals?? (the Ethics Code)

Response: Yes. The Ethics Approval Codes issued by Tehran University of Medical Sciences are all in this format. It might be better to keep it in capital.

Restate to reflect true meaning (the statistical analysis section)

Response: We revised the paragraph on statistical analysis to reflect true meaning (page 5, last paragraph). We hope it is quite clear now.

antibiotics taken

Response: Revised (page 8, first paragraph).

after the emergence of the pandemic (in discussion).

Response: Revised (page 12, first paragraph).

Check spacing of paragraphs (in discussion part).

Response: Checked and revised.
