# Supplementary material for: A cross-sectional study on COVID-19-related changes in self-medication with antibiotics
Source: PLoS One. 2022 Jun 14;17(6):e0269782. doi: 10.1371/journal.pone.0269782 (PMC9197057; doi:10.1371/journal.pone.0269782)
Supplement: Attachment — Submitted filename: Response to Reviewers.docx [file pone.0269782.s006.docx]

Response to Reviewers

Additional Editor Comments:

**Comment:** According to author "The present study was carried out in a large professional dental clinic, which is a referral center
for all types of oral and dental conditions in a large city in Iran. Also, all the information was
extracted and recorded by one dentist, which was another strength of this study. However, the
cross-sectional design was the main limitation of the present study"

I request to provide a separate heading under the limitation of study and write my in detail about the limitation of the study

**Response:** We thank the editor for this comment. A separate heading and paragraph about the limitations of the study was added according to the comment (page 13, paragraph 2).
