# Supplementary material for: A cross-sectional study on COVID-19-related changes in self-medication with antibiotics
Source: PLoS One. 2022 Jun 14;17(6):e0269782. doi: 10.1371/journal.pone.0269782 (PMC9197057; doi:10.1371/journal.pone.0269782)
Supplement: Attachment — Submitted filename: Response to Reviewers 2_MK.docx [file pone.0269782.s007.docx]

Response to Reviewers

Additional Editor Comments:

**Comment:** Additional Editor Comments:

1. Please write in detail the limitation of the study design, methodological limitation

Kindly explain why we cannot able to assess causation and state recommendation.

Kindly explain self-report nature of the study and state recommendation.

Response: We thank the editor for this comment. The paragraph about the limitations of the study was revised according to the comment, and the requested aspects were covered (page 13, paragraph 2).

“The cross-sectional design was the main limitation of the present study, as this study type is unable to assess causation. Cross-sectional studies are used to look for the presence or absence of an outcome as well as the presence or absence of an exposure at a certain period. Cross-sectional studies can be conducted without the requirement for follow-up, making them more cost-effective. Because both the outcome and the exposure are investigated at the same time, the temporal link between the outcome and the exposure cannot be ascertained in cross-sectional research (30). Because a chronological sequence cannot be established, the cross-sectional study cannot be utilized to infer causality. This type of study is still used to obtain descriptive data about a population's disease/outcome burden or to assess background exposure rates (31).

Another limitation was the self-report nature of the study, which induces the risk of recall bias. Moreover, it increases the possibility of giving favorable responses by the respondents, which is called “social desirability” (32). Thus, the results may be an optimistic estimation of the real situation. In questionnaire research, there's a chance of misunderstandings and errors (33). That our questionnaire was a part of routine medical and dental records of the patients reduces the likelihood of this bias, however. Anyway, the results should be interpreted cautiously.”

2. Do this study have Prevalence-incidence bias? If yes then explain.

Please indicate potential bias in the study and explain in details

Response: We thank the editor for raising this point. Prevalence-incidence bias occurs when individuals with severe or mild disease are excluded, leading to an error in the estimated association between an exposure and an outcome. It seems that we do not have prevalence-incidence bias in our study because our clinic was a referral center and patients came in with any degree of conditions.
